# Oncogenic Processes

**DOI:** 10.1155/2014/879013

**Published:** 2014-01-16

**Authors:** Rita de Cassia Stocco, Franco Peppino Roperto, Lubna Nasir, Marcelo Palma Sircili

**Affiliations:** ^1^Laboratório de Genética, Instituto Butantan, Secretaria de Estado da Saúde, Avenida Vital Brasil, 1500 Butantã, 05503-900 São Paulo, SP, Brazil; ^2^Department of Biology, Naples University Federico II, Via Mezzocannone 16, 80134 Naples, Italy; ^3^Institute of Infection, Immunity and Inflammation, College of Medical, Veterinary and Life Sciences, MRC-University of Glasgow Centre for Virus Research, Bearsden Road, Glasgow G61 1QH, UK

The relevance of cancer to human health is extremely high and the efforts to understand and control the processes related to the oncogenic modifications that take place in cells have increased, dramatically, in the past few decades [1–3]. The special issue presents a broad view of this subject.

Actually, oncogenic process was discussed by the authors that contributed to the Special Issue and have clearly demonstrated that this process involves the action of different agents on the cell [1, 4–7].

The major contribution achieved was the diversity of the approaches; the studies have brought together important aspects of cell transformation and also concerted efforts directed towards therapeutic procedures. The editors hope that this issue can help partially to the complex problems involved in these processes.

When we consider the etiology of tumor cells, papillomaviruses come immediately to mind: the relevance of HPVs (human papillomavirus) in female cervical cancer has obtained the priority in the virus-cancer relationship discussion [1, 2, 4, 8–10]. Its incidence turns it in a worldwide human health problem. In this volume, we have the opportunity to study persistence or clearance in HPV infection in order to allow the identification of risk groups, cofactors, and strategies for prevention of cervical cancer. At the same time, the issue presents results of research on less common HPV types that might be involved in cervical lesions and that some of these variants can be found in B-cell and T-cell epitopes [11–15].

Animal models have played a contributions to the understanding of oncogenic processes [5, 10, 16–26]. One of them deserves special attention: *Bovine papillomavirus*. This group of viruses is directly related to serious clinical consequences in bovine, namely papillomatosis, esophagus tract carcinoma, and urinary bladder carcinoma leading to animal death and in equids, sarcoid tumours that presents similar results [10, 18, 27]. These diseases lead to dramatic economic hazards. BPV animal model has been studied in several aspects, as illustrated in the different papers included in this issue, since many aspects of bovine papillomavirus infection and pathogenesis remain still to be clarified [28]. The oncogenesis process is mainly associated with different viral oncoprotein expressions, which are involved in cell transformation [7, 12, 19, 29]. The accuracy of diagnostic processes and the distribution of the different viral types [14, 26, 30–32] indicate which types and variants deserve the special attention [8, 9, 25, 33, 34]. The expression and characterization of recombinant viral oncoproteins are required to obtain biotechnological products as antibodies and potential vaccines [9, 15, 35–37]. Further studies show association of oncoproteins and cell compounds, as PDGF*β* receptor and their actions in transformation of epithelial cell to mesenchymal cell as well as epithelial carcinogenesis of the urinary bladder [17, 33, 38]. The oncoproteins also act on host cell chromatin, in epithelial and blood cells [18, 23, 27, 39]. The virus DNA sequences have been described in semen, urine, and other non conventional host cells [10, 17, 20, 21, 23, 29, 33]. However, the initial event of the malign process remains not determined.

Chromosome aberrations can be detected in very diverse neoplastic process; bovine leukemia virus (BLV) was investigated and reported presenting chromosome breakage in lymphocytes [16] and in the central nervous system (CNS) of cattle with neurological syndrome. While in papillomavirus, the chromosome aberrations occur at random, in tumor cells, virus sequences can be integrated in specific sites or the malign process can be related to products as in leukemia, fusion protein RUNX1/ETO that is generated by the chromosomal translocation t(8; 21) [40–43].

The oncogenic process can have another trigger, not specifically chromatin lesions. Gap junctions are communicating junctions which are important for tissue homeostasis, and their disruption is involved in carcinogenic process; connexin 43 deficiency is a clear example [44–46]. Cell surface proteins are related as targets for the beginning of the cancer development and consequently for cancer therapy or diagnosis [47–50]. These aspects have been well discussed in oncogenic processes; the expression of different genes in breast tumors, alternative TrkAIII splice variant expressed by advanced stage human neuroblastomas (NBs).

In a final evaluation, the papers pointed very clearly the complexity of the events leading to cancer. This complexity, obviously, reaches the diagnosis and therapy possibilities. In the ongoing studies, molecules are being analyzed to create ways to interfere with the tumor cell. In the special issue, viral oncoproteins were also considered through bioinformatics approach. The volume includes a detailed review of a purified peptide from South American rattle snake, a venom chemical crotamine [51–53] that is being discussed as a vector to reach the malign cell.

Our major aim in organizing this volume was to emphasize that the first step in oncogenic process is until now under discussion and therefore is the key for prophylaxis, diagnosis, and therapy. An attractive possibility is that the key remains in any alteration in the natural history of a cell; any cell, as any organism, has beginning, development, ageing, and death. When this route is modified, due to a large range of events, this cell can follow a different way and compete with its similar tissue cells, and, when this occurs in advantage, tumor cells win and the cancer happens.

The special issue (*oncogenic processes*) is celebrating the 80th birthday of Prof Dr. Willy Beçak, former Director of Instituto Butantan and remarkable researcher in cancer etiology studies and in the developing of vaccines.

We hope that this special issue (*oncogenic processes*) can contribute to this scientific area, bringing to readers accurate data and important discussions about this subject; but, mainly, we hope that this special issue will initiate new discussions relating to the elucidation of mechanisms and oncogenic process.

*Rita de Cassia Stocco* *Franco Peppino Roperto* *Lubna Nasir* *Marcelo Palma Sircili*
